# Association between the triglyceride-glucose index and major adverse cardiovascular events in patients with chronic kidney disease stages 3–4

**DOI:** 10.1038/s41598-025-14057-1

**Published:** 2025-08-05

**Authors:** Fan Zhu, Wenyuan Gan, Huihui Mao, Sheng Nie, Xingruo Zeng, Wenli Chen

**Affiliations:** 1 Department of Nephrology, Tongji Medical College, The Central Hospital of Wuhan, Huazhong University of Science and Technology, Wuhan, Hubei, 430014 China; 2 Division of Nephrology, State Key Laboratory of Organ Failure Research, National Clinical Research Center for Kidney Disease, Nanfang Hospital, Medical University, 1838 North Guangzhou Avenue, Southern, Guangzhou, 510515 China

**Keywords:** TyG index, MACE, Diabetes, All-cause mortality, Endocrine system and metabolic diseases, Kidney diseases

## Abstract

The association between the triglyceride–glucose (TyG) index and the risk of major adverse cardiovascular events (MACE) in patients with chronic kidney disease (CKD) stages 3–4 has not been extensively studied. This study aims to investigate the relationship between baseline TyG index and MACE risk in a CKD stage 3–4 population. This study utilized data from the 2000–2022 China Renal Data System. Multivariate regression analysis models were constructed to explore the association between baseline TyG index and MACE. We employed restricted cubic splines to examine potential nonlinear correlations between these variables. Subgroup analyses were performed for different clinical endpoints. A total of 48,935 participants with CKD stages 3–4 were enrolled, with a mean TyG index of 8.88 [8.45, 9.38]. Participants were divided into quartiles based on TyG index values (quartile thresholds: 8.33, 8.78, 9.24). The overall prevalence of MACE was 15.90%. Multivariate Cox regression indicated a significant association between TyG quartiles and MACE occurrence, with hazard ratios (HR) of 1.08 (95% CI: 1.01–1.15, *p* = 0.016) for Quartile 1 and 1.12 (95% CI: 1.05–1.20, *p* = 0.001) for Quartile 4. Restricted cubic spline analysis revealed a nonlinear relationship between TyG index and MACE risk (P for nonlinearity < 0.001) among individuals with CKD stages 3–4. In patients with CKD stages 3–4, the TyG index shows a nonlinear association with the risk of MACE and all-cause mortality. These findings suggest that both elevated and reduced TyG index levels may increase the likelihood of MACE and all-cause mortality.

## Introduction

Chronic kidney disease (CKD) is characterized by structural and functional damage to the kidneys, leading to a range of systemic complications and multi-organ dysfunction, reducing patient quality of life and severely impacting survival. Epidemiological studies indicate that CKD affects 850 million people worldwide^1^with stage 3 CKD being the most prevalent (the prevalence of each CKD stage is estimated at 3.5% for stage 1, 3.9% for stage 2, 7.6% for stage 3, 0.4% for stage 4, and 0.1% for stage 5)^2^. Major adverse cardiovascular events (MACE) are a primary cause of poor outcomes in CKD patients, accounting for approximately one-third of deaths among these individuals due to cardiovascular complications and related MACE. Cardiovascular risk has thus become a major health burden for CKD patients, posing significant challenges to clinical management and survival outcomes^3–6^.

In this study, we specifically focused on patients with CKD stages 3–4. Individuals with CKD stages 1–2 generally have only mild reductions in kidney function, often with low cardiovascular event rates and a lower prevalence of metabolic complications^7^which may limit the clinical utility of risk prediction in these groups. On the other hand, CKD stage 5 and ESRD patients frequently have profoundly altered metabolic profiles and may be undergoing renal replacement therapy, introducing additional confounding factors that can obscure the relationship between metabolic indices and cardiovascular risk. By focusing on stages 3–4, we sought to examine the association between the TyG index and cardiovascular risk in a clinically relevant population at increased risk, while minimizing potential confounding from extreme disease states or treatment effects.

As kidney function declines, patients experience imbalances in acid-base homeostasis, electrolytes, hormone secretion, and metabolism. Insulin resistance can occur in varying degrees across CKD stages, from early CKD to end-stage renal disease (ESRD), regardless of diabetes status^8–10^. Compared to the classic hyperinsulinemic-euglycemic clamp technique, the triglyceride-glucose (TyG) index, calculated using fasting blood glucose (FBG) and triglycerides (TG), has emerged as a low-cost, accessible, and reliable proxy for assessing insulin resistance^11^. Insulin resistance is associated with asymptomatic atherosclerosis and coronary artery disease and is considered a predictor of cardiovascular disease (CVD), primarily studied in populations with diabetes and obesity^12–15^.

Given the kidney’s role in metabolizing toxins and regulating glucose-insulin dynamics, applying conventional TyG research directly to CKD populations may not be appropriate. Studies suggest that in ESRD populations, the TyG index predicts 1-year MACE risk; however, evidence is limited regarding its predictive value in populations with mild to moderate kidney dysfunction, lacking large-scale research support^16^. This study, a large-scale retrospective analysis, leverages data from a multicenter cohort study to evaluate the predictive value of the TyG index for assessing MACE risk in Chinese patients with CKD stages 3–4.

## Methods

### Study population

**Fig. 1:**
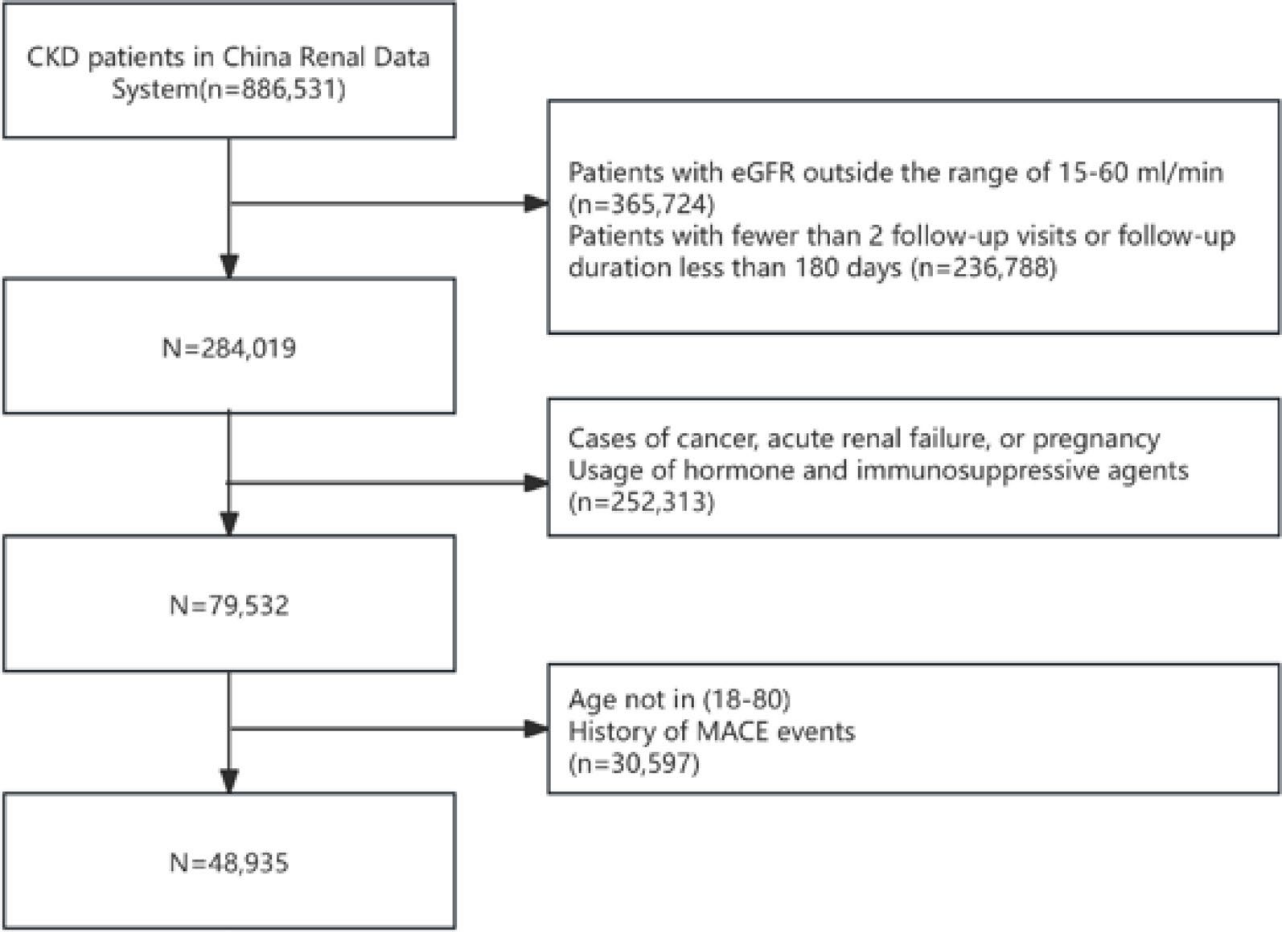
Patient screening flowchart.

Our research is a multicenter retrospective cohort study utilizing the China Renal Data System (CRDS), targeting all individuals diagnosed with CKD during their hospital stays or outpatient visits between January 1, 2000, and December 31, 2022. As shown in Fig. 1, within the specified time period, a total of 886,531 patients were considered for CKD diagnosis in the CRDS. After applying the exclusion criteria, 48,935 patients were included in the final analysis.

Chronic kidney disease (CKD) staging in this study was determined based on the estimated glomerular filtration rate (eGFR) calculated using the CKD-EPI equation. Albuminuria data were not routinely available for all participants in the China Renal Data System; therefore, albuminuria was not included in the primary CKD staging. This approach is consistent with the KDIGO guidelines, which define CKD G-stage primarily by eGFR, while albuminuria is recommended for risk stratification, and patients with baseline estimated glomerular filtration rate (eGFR) between 15 and 60 ml/min were selected. These patients were categorized as having stage 3–4 CKD, according to the staging guidelines established by the Kidney Disease Outcomes Quality Initiative (KDOQI) in 2002. The inclusion criteria were: (1) a confirmed diagnosis of chronic kidney disease at stages 3a, 3b, or 4; (2) a minimum of two outpatient visits or hospitalizations with at least two assessments of kidney function. The exclusion criteria included: (1) patients who had undergone hemodialysis (HD), peritoneal dialysis (PD), or kidney transplantation; (2) patients aged 18–80 years; (3) patients with concurrent conditions such as pregnancy, malignant neoplasms, or acute active bleeding disorders; (4) patients experiencing an episode of acute kidney injury (AKI); (5) patients with systemic autoimmune diseases or those receiving hormone replacement therapy, immunosuppressants, or cytotoxic medications at baseline or in the past three months; (6) patients with a history of MACE.

### Variables and covariates collection

The data used in this study were sourced from the CRDS database, which contains information from multiple centers, consolidated after de-identification of patient-sensitive data. The collected information included demographic variables such as age, gender, comorbidities, medical history, blood pressure, and body mass index (BMI). Laboratory examinations provided data on hemoglobin (Hb), serum creatinine (Scr), blood urea nitrogen (BUN), serum uric acid (UA), albumin (Alb), calcium, potassium, phosphorus levels, estimated glomerular filtration rate (eGFR), high-sensitivity C-reactive protein (hs-CRP), triglycerides (TG), cholesterol, glucose, high-density lipoprotein (HDL-C), and low-density lipoprotein (LDL-C). Medication records included whether patients were prescribed renin-angiotensin system inhibitors (RASI), which include angiotensin-converting enzyme inhibitors (ACEI) or angiotensin receptor blockers (ARB). It also documented insulin use, lipid-lowering agents (LLA), including statins, fibrates (e.g., fenofibrate), and ezetimibe. Diuretic use was recorded for patients taking long-term oral diuretics, whereas those receiving a single intravenous dose during a temporary hospital stay were excluded.

Hypertension was defined as three or more measurements of systolic blood pressure (SBP) equal to or greater than 140 mmHg or diastolic blood pressure (DBP) equal to or greater than 90 mmHg, or the use of antihypertensive medication (if medication use began prior to study inclusion). Diabetes was defined based on the following factors: the use of oral hypoglycemic medications or insulin (if medication use began prior to study inclusion), or glycated hemoglobin (HbA1c) levels equal to or greater than 6.5% upon admission.

Estimated glomerular filtration rate (eGFR) was calculated using the CKD-EPI (Chronic Kidney Disease Epidemiology Collaboration) 2009 Equation^17^. For females, if serum creatinine (Scr) ≤ 0.7 mg/dL, eGFR = 144 × (Scr/0.7) ^–0.329 × (0.993) ^Age; if Scr > 0.7 mg/dL, eGFR = 144 × (Scr/0.7) ^–1.209 × (0.993) ^Age. For males, if Scr ≤ 0.9 mg/dL, eGFR = 141 × (Scr/0.9) ^–0.411 × (0.993) ^Age; if Scr > 0.9 mg/dL, eGFR = 141 × (Scr/0.9) ^–1.209 × (0.993) ^Age.

Age was expressed in years, and serum creatinine was measured in mg/dL. Race coefficients were not included, as all participants were of Asian ethnicity.

The TyG index was calculated using the following formula:

$${\text{TyG index}}\,=\,{\text{ln }}\left( {\frac{{{\text{T}}G\left( {mg/dl} \right) \times {\text{Glucose}}\left( {{\text{m}}g/dl} \right)}}{2}} \right)$$

To ensure the accuracy of the TyG index, glucose and TG levels were obtained from the same batch of blood samples. Additionally, the Atherogenic Index of Plasma (AIP) was calculated using the formula:

$$AIP = \:{\text{log}}\left( {\frac{{TG\left( {mmol/l} \right)}}{{{\text{H}}DL - c\left( {mmol/l} \right)}}} \right)$$

To ensure accuracy and consistency, all data entries were validated by two independent authors.

### Outcomes and follow-up

The primary endpoint, major adverse cardiovascular events (MACE), was defined as a composite of acute myocardial infarction (AMI), hemorrhagic or ischemic stroke, cardiovascular-related death, rehospitalization due to unstable angina, or coronary revascularization surgery. This definition is consistent with recent large-scale clinical studies and is supported by the latest international guidelines, including the 2025 ACC/AHA/ACEP/NAEMSP/SCAI Guideline for the Management of Patients With Acute Coronary Syndromes and the 2023 ESC Guidelines for the management of acute coronary syndromes, both of which endorse a more flexible approach to defining MACE in contemporary research settings^18,19^. Acute myocardial infarction was diagnosed according to the Fourth Universal Definition of Myocardial Infarction^20^. Cardiovascular-related deaths were recorded by linking the CRDS with the China National CDC. Rehospitalization due to unstable angina or coronary revascularization was determined using patient hospitalization records, electrocardiogram results, and clinical progression, as well as related surgical interventions. Given that B-type natriuretic peptide (BNP) and N-terminal pro-BNP (NT-proBNP) levels are often elevated in CKD patients due to impaired metabolism, and that the evaluation of heart failure symptoms may involve considerable subjectivity, this study used MACE outcomes encompassing the four components described above, while heart failure was analyzed separately. Additionally, data on all-cause mortality were collected and analyzed. Follow-up for each participant began at the date of study entry and ended at the earliest occurrence of a primary endpoint event (MACE), death, last available clinical visit, loss to follow-up, or the end of the study period (December 31, 2022).

## Results

Of the 48,935 patients included in the study, 29,485 (60.3%) were male, with a mean age of 65.15 years [IQR 54.19, 73.26]. The overall follow-up time was 28.4 [IQR 14.0, 51.6] months. During follow-up, a total of 7,759 MACE events (15.9%) occurred, including 1,483 cases (3.03%) of unstable angina, 1,750 cases (3.58%) of non-fatal myocardial infarction, 2,938 cases (6.00%) of stroke, 57 cases (0.12%) of coronary revascularization, and 3,204 cases (6.54%) of cardiovascular-related death. For MACE event recording, concurrent occurrences of different MACE events during the same hospitalization were recorded as a single event. Among all recorded deaths, cardiovascular-related deaths accounted for 3,204 cases (72.3%), while non-cardiovascular-related deaths accounted for 27.7%. The median TyG and FBG were 1.53 [IQR 1.08, 2.25] and 5.58 [IQR 4.87, 7.15], respectively. The TyG index ranged from 4.66 to 13.74 [IQR 8.40, 9.36]. Patients were divided into four quartiles based on TyG levels, as shown in Table 1. Apart from urine ACR, SBP, DBP, follow-up time, and Charlson score, which followed a normal distribution and were presented as mean ± standard deviation, all other variables were skewed and presented as median (IQR).

Table 1 summarizes baseline characteristics according to TyG index quartiles. Significant differences were observed across quartile groups for most demographic, clinical, and laboratory variables, including age, sex, blood pressure, BMI, prevalence of hypertension and diabetes, eGFR, and key laboratory indices such as fasting glucose, triglycerides, lipid profile, and proteinuria (all *p* < 0.001). The use of medications—including insulin, RAS inhibitors, lipid-lowering agents, and diuretics—also varied significantly between quartiles. All group comparisons were performed using ANOVA, Kruskal-Wallis, or Chi-square tests, as appropriate.

**Table 1:** Baseline clinical characteristics of patients according to tertiles of triglyceride-glucose index.

TyG index	Overall (8.40–9.36)	Q1 (7.81, 8.26)	Q2 (8.52, 8.74)	Q3 (8.96, 9.21)	Q4 (9.54, 10.18)	*p* value
N	48,935	12,236	12,232	12,233	12,234	
Follow-up duration	28.4 [14.0, 51.6]	29.0 [14.5, 52.2]	30.2 [14.9, 53.0]	29.8 [15.2, 53.5]	27.5 [13.1, 50.4]	< 0.001
Sex (Male, %)	29,485 (60.3)	7850 (64.2)	7490 (61.2)	7213 (59.0)	6932 (56.7)	< 0.001
Age, year	65.15 [54.19, 73.26]	66.70 [54.70, 74.41]	66.20 [54.96, 74.06]	64.70 [54.08, 72.83]	63.27 [53.38, 71.47]	< 0.001
BMI, kg/m^2^	24.22 [24.14, 24.97]	24.13 [23.04, 24.97]	24.26 [24.00, 25.03]	24.31 [24.22, 25.14]	24.40 [24.25, 25.21]	< 0.001
Hypertension (%)	23,529 (48.1)	5584 (45.6)	5627 (46.0)	5944 (48.6)	6374 (52.1)	< 0.001
DM (%)	16,388 (33.5)	2813 (23.0)	2892 (23.6)	3941 (32.2)	6742 (55.1)	< 0.001
RASI (%)	19,236 (39.3)	4439 (36.3)	4534 (37.1)	4835 (39.5)	5428 (44.4)	< 0.001
Diuretics (%)	12,877 (26.3)	3379 (27.6)	3024 (24.7)	3091 (25.3)	3383 (27.7)	< 0.001
LLA (%)	18,115 (37.0)	3892 (31.8)	3982 (32.6)	4539 (37.1)	5702 (46.6)	< 0.001
Insulin (%)	11,041 (22.56)	1916 (15.66)	1769 (14.46)	2457 (20.09)	4899 (40.04)	< 0.001
Hb, g/l	124.33 (23.35)	120.71 (23.66)	124.04 (23.29)	125.88 (23.08)	126.68 (22.91)	< 0.001
Alb, g/l	39.30 [34.90, 43.00]	38.30 [34.20, 42.00]	39.60 [35.40, 43.10]	40.00 [35.60, 43.70]	39.30 [34.40, 43.20]	< 0.001
eGFR, ml/min/(1.73.m^2^)	44.25 [32.12, 52.77]	44.63 [32.28, 53.09]	44.72 [32.76, 53.01]	44.15 [32.06, 52.62]	43.55 [31.50, 52.30]	< 0.001
Scr, umol/l	1.52 [1.29, 1.99]	1.52 [1.29, 1.99]	1.50 [1.28, 1.96]	1.53 [1.28, 2.00]	1.54 [1.30, 2.01]	< 0.001
Bun, mmol/l	8.40 [6.60, 11.15]	8.36 [6.56, 11.10]	8.19 [6.45, 10.80]	8.40 [6.58, 11.08]	8.75 [6.90, 11.60]	< 0.001
ACR, mg/g	858.11 ± 2252.35	680.12 ± 1384.53	682.84 ± 1665.22	880.28 ± 2497.19	1189.21 ± 3032.40	< 0.001
Hs-CRP	3.66 [1.30, 11.35]	3.71 [1.30, 11.68]	3.50 [1.30, 11.10]	3.50 [1.30, 10.70]	3.90 [1.40, 11.80]	< 0.001
LDL-c, mmol/l	2.74 [2.08, 3.41]	2.41 [1.82, 3.01]	2.72 [2.10, 3.31]	2.85 [2.25, 3.59]	2.86 [2.22, 3.73]	< 0.001
HDL-c, mmol/l	0.98 [0.87, 1.32]	1.16 [0.93, 1.44]	1.11 [0.91, 1.35]	1.05 [0.86, 1.28]	1.00 [0.81, 1.22]	< 0.001
TC, mmol/l	4.62 [3.81, 5.58]	4.16 [3.42, 4.97]	4.49 [3.73, 5.32]	4.79 [3.98, 5.71]	5.17 [4.25, 6.26]	< 0.001
TG, mmol/l	1.53 [1.08, 2.25]	0.90 [0.73, 1.09]	1.31 [1.13, 1.51]	1.87 [1.55, 2.21]	2.93 [2.17, 3.98]	< 0.001
Glucose, mmol/l	5.58 [4.87, 7.15]	4.94 [4.42, 5.58]	5.29 [4.76, 6.00]	5.78 [5.08, 7.04]	8.07 [5.96, 11.79]	< 0.001
UA, mmol/l	441.00 [367.00, 533.00]	434.90 [356.00, 522.00]	436.22 [363.00, 528.00]	449.00 [375.00, 538.00]	451.00 [376.00, 543.18]	< 0.001
K, mmol/l	4.18 [3.82, 4.55]	4.17 [3.82, 4.54]	4.17 [3.82, 4.52]	4.19 [3.84, 4.55]	4.19 [3.82, 4.57]	0.056
Na, mmol/l	140.70 [138.00, 143.00]	141.00 [138.20, 143.00]	141.00 [138.70, 143.00]	141.00 [138.40, 143.00]	140.00 [137.00, 142.00]	< 0.001
Cl, mmol/l	105.00 [102.00, 108.00]	105.30 [102.35, 108.30]	105.00 [102.40, 108.00]	105.00 [102.00, 108.00]	104.00 [100.90, 107.06]	< 0.001
P, mmol/l	1.15 [1.00, 1.31]	1.13 [0.99, 1.30]	1.14 [0.99, 1.30]	1.15 [1.00, 1.31]	1.17 [1.01, 1.33]	< 0.001
Ca, mmol/l	2.15 [1.96, 2.30]	2.15 [1.96, 2.29]	2.14 [1.96, 2.30]	2.14 [1.95, 2.31]	2.16 [1.96, 2.33]	< 0.001
SBP, mmHg	124.27 ± 28.54	123.85 ± 28.03	123.29 ± 28.38	123.59 ± 27.95	126.35 ± 29.68	< 0.001
DBP, mmHg	78.28 ± 27.22	77.32 ± 22.42	78.09 ± 28.97	78.56 ± 27.86	79.15 ± 29.04	< 0.001
Proteinuria (%)						< 0.001
0	21,370 (43.7)	5978 (48.9)	5796 (47.4)	5226 (42.7)	4370 (35.7)	
0.5	4353 (8.9)	1112 (9.1)	1195 (9.8)	1092 (8.9)	954 (7.8)	
1	8240 (16.8)	2116 (17.3)	2068 (16.9)	2045 (16.7)	2011 (16.4)	
2	8399 (17.2)	1899 (15.5)	1931 (15.8)	2137 (17.5)	2432 (19.9)	
3	5902 (12.1)	1047 (8.6)	1104 (9.0)	1544 (12.6)	2207 (18.0)	
4	671 (1.4)	84 (0.7)	138 (1.1)	189 (1.5)	260 (2.1)	
AIP	0.16 [-0.04, 0.38]	-0.11 [-0.25, 0.04]	0.07 [-0.04, 0.19]	0.25 [0.12, 0.37]	0.47 [0.31, 0.66]	< 0.001
TyG index	8.85 [8.40, 9.36]	8.08 [7.81, 8.26]	8.63 [8.52, 8.74]	9.08 [8.96, 9.21]	9.79 [9.54, 10.18]	< 0.001
MACE (%)	7759 (15.9)	1989 (16.3)	1830 (15.0)	1888 (15.4)	2052 (16.8)	< 0.001
Charlson.score (%)	4.19 (1.64)	4.16 (1.67)	4.14 (1.54)	4.15 (1.60)	4.30 (1.75)	< 0.001
All-cause death (%)	4434 (9.06)	1290 (10.54)	1094 (8.94)	992 (8.11)	1058 (8.65)	< 0.001

Data Presentation: Data are presented as mean (SD), n (%), or median [25th − 75th percentile].

Model 1: Unadjusted.

Model 2: Adjusted for age and sex.

Model 3: Adjusted for age, sex, systolic blood pressure, diastolic blood pressure, BMI, comorbidities (hypertension, diabetes mellitus, heart failure), laboratory test results (Hb, Alb, UA, eGFR, Scr, BUN, ACR, hs-CRP, LDL-C, HDL-C, TC, K, Na, Cl, P, Ca, Proteinuria), and medications (insulin, RAS inhibitors, lipid-lowering agents [LLA], diuretics).

### Association between TyG index and MACE

**Fig. 2:**
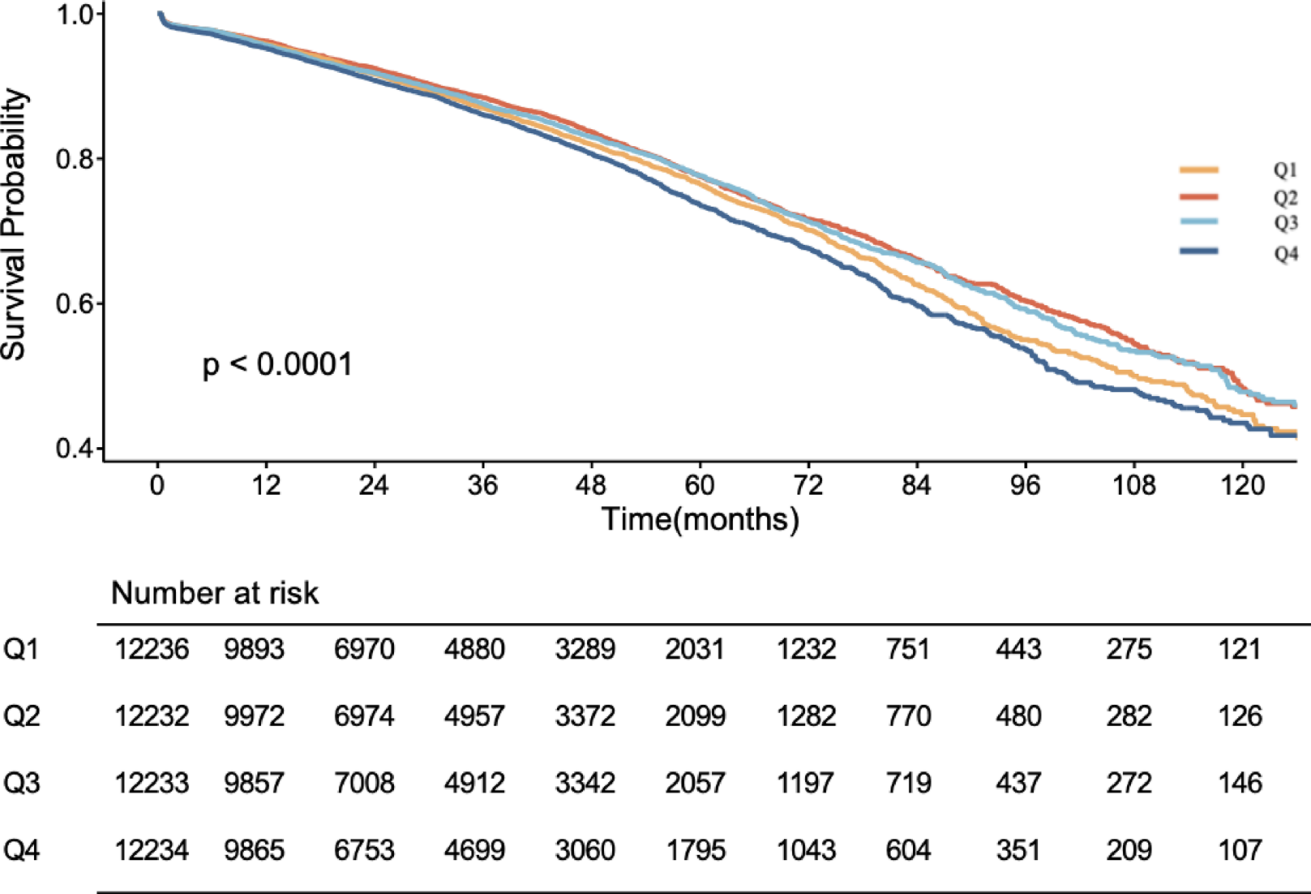
Survival outcomes by different TyG groups: MACE according to quartiles of TyG index.

The average follow-up time for all participants was 35.36 months, during which 7,759 MACE events (15.9%) occurred. Time-to-event analyses are presented in Fig. 2. The Kaplan–Meier curve indicated that quartiles of the TyG index (especially Q4) were associated with a higher risk of MACE (log-rank test, *p* < 0.001). As the TyG index increased, the number and percentage of MACE events in quartiles Q1 to Q4 were as follows: Q1 (1,989, 16.3%), Q2 (1,830, 15.0%), Q3 (1,888, 15.4%), Q4 (2,052, 16.8%). The incidence of MACE was lowest in Q2, and thus Q2 was used as the reference group for subsequent analyses.

Table 2 shows the hazard ratios (HR) and 95% confidence intervals (CI) of the continuous TyG index and TyG quartiles (Q1 to Q4) with MACE across different models (model 1, model 2, and model 3). The continuous TyG index was significantly associated with MACE in the unadjusted model (model 1) (HR: 1.04, 95% CI: 1.01–1.06). For the TyG quartiles, compared to Q2, Q1 (HR: 1.11, 95% CI: 1.04–1.18) and Q4 (HR: 1.20, 95% CI: 1.13–1.28) were significantly associated with MACE, while Q3 (HR: 1.04, 95% CI: 0.98–1.11) did not show a significant positive correlation with MACE (*p* = 0.234).

**Table 2:** The association between various TyG index and MACE.

TyG	Model1	*p* value	Model2	*p* value	Model3	*p* value
	HR (95%CI)		HR (95%CI)		HR (95%CI)	
Continues	1.04 (1.01–1.06)	0.002	1.08 (1.05–1.10)	0.000	1.01 (0.99–1.04)	0.266
Quartiles						
Q1	1.11 (1.04–1.18)	0.002	1.09 (1.02–1.16)	0.008	1.08 (1.01–1.15)	0.016
Q2	Ref	Ref	Ref	Ref	Ref	Ref
Q3	1.04 (0.98–1.11)	0.234	1.06 (1.01–1.17)	0.007	1.06 (0.98–1.14)	0.194
Q4	1.20 (1.13–1.28)	< 0.001	1.33 (1.25–1.42)	< 0.001	1.12 (1.05–1.20)	0.001

Model 1: Unadjusted.

Model 2: Adjusted for age and sex.

Model 3: Adjusted for age, sex, systolic blood pressure, diastolic blood pressure, BMI, comorbidities (hypertension, diabetes mellitus, heart failure), laboratory test results (Hb, Alb, UA, eGFR, Scr, BUN, ACR, hs-CRP, LDL-C, HDL-C, TC, K, Na, Cl, P, Ca, Proteinuria), and medications (insulin, RAS inhibitors, lipid-lowering agents [LLA], diuretics).

In model 2, adjusted for gender and age, the continuous TyG index was significantly associated with MACE (HR: 1.08, 95% CI: 1.05–1.10). Compared to Q2, Q1 (HR: 1.09, 95% CI: 1.02–1.16), Q3 (HR: 1.06, 95% CI: 1.01–1.17), and Q4 (HR: 1.33, 95% CI: 1.25–1.42) were all significantly associated with MACE. In the fully adjusted model (model 3), the continuous TyG index was not significantly associated with MACE (HR: 1.01, 95% CI: 0.99–1.04). Compared to Q2, Q1 (HR: 1.08, 95% CI: 1.01–1.15) and Q4 (HR: 1.12, 95% CI: 1.05–1.20) were significantly associated with MACE, while Q3 (HR: 1.06, 95% CI: 0.98–1.14) did not show a significant positive correlation with MACE (*p* = 0.194).

**Fig. 3:**
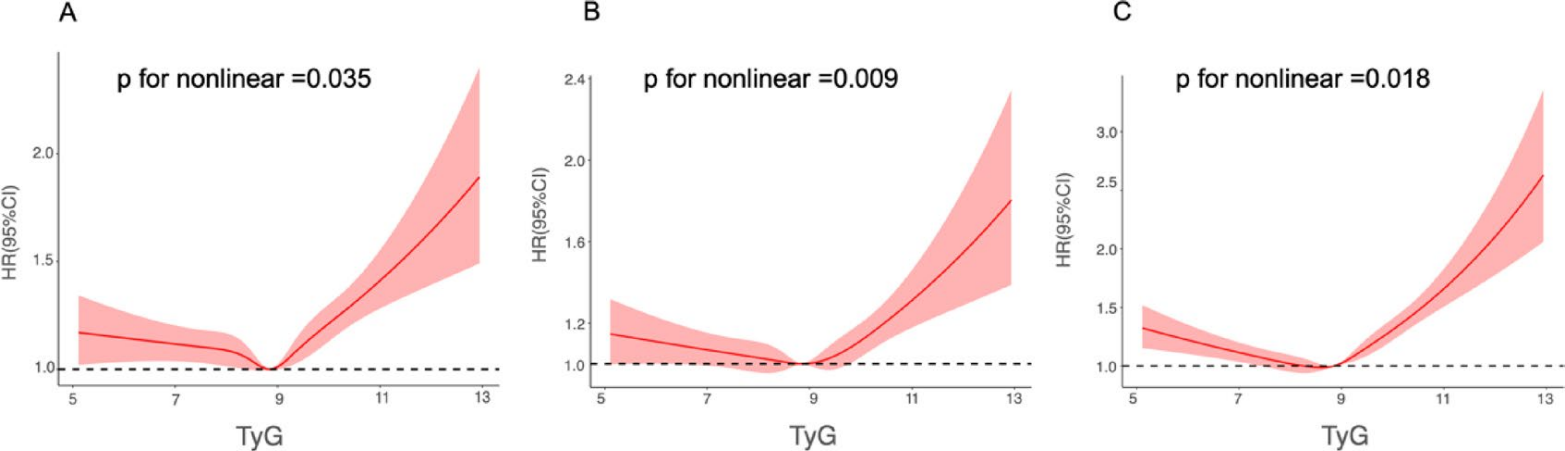
Restricted cubic spline curves of the association between TyG and MACE. (**A**). Unadjusted model. (**B**). adjusted for age and sex. (**C**). fully adjusted model. Hazard ratios are indicated by solid red lines and 95% CIs are indicated by shaded areas. CI, confidence interval; TyG, triglyceride-glucose index.

The restricted cubic spline (RCS) plots presented in Fig. 3 also demonstrated a nonlinear relationship between the TyG index and MACE in models 1 (Fig. 3A), 2 (Fig. 3B), and 3 (Fig. 3C) (p for nonlinearity < 0.001). The RCS plots exhibited a U-shaped curve.

### Association between TyG index and All-Cause mortality

In the same study population, when the outcome was all-cause mortality, the average follow-up period was 39.23 months, with a total of 8,480 (17.3%) all-cause death events occurring. Time-to-event analyses are shown in Supplementary Fig. 1. The Kaplan–Meier curve suggested that TyG index quartiles (Q1, Q4) were associated with a higher risk of all-cause death (log-rank test, *p* < 0.001). Quartile 2 was used as the reference group for analysis, and Supplementary Table 1 presents the hazard ratios (HR) and 95% confidence intervals (CI) of the continuous TyG index and TyG quartiles (Q1–Q4) for all-cause death across different models (model 1, model 2, and model 3). In the fully adjusted model (model 3), the continuous TyG index had an HR (95% CI) of 0.96 (0.93–0.98), *p* < 0.001. For quartiles Q1 and Q4, the HRs (95% CI) were 1.16 (1.09–1.23), *p* < 0.001 and 1.07 (1.02–1.14), *p* = 0.031, respectively. Additionally, the restricted cubic spline (RCS) curves shown in Supplementary Fig. 2 indicated a nonlinear relationship between the TyG index and all-cause death in models 1 (Supplementary Fig. 2A), 2 (Supplementary Fig. 2B), and 3 (Supplementary Fig. 2C) (p for nonlinearity < 0.001), with a U-shaped curve.

### Subgroup analyses

**Fig. 4:**
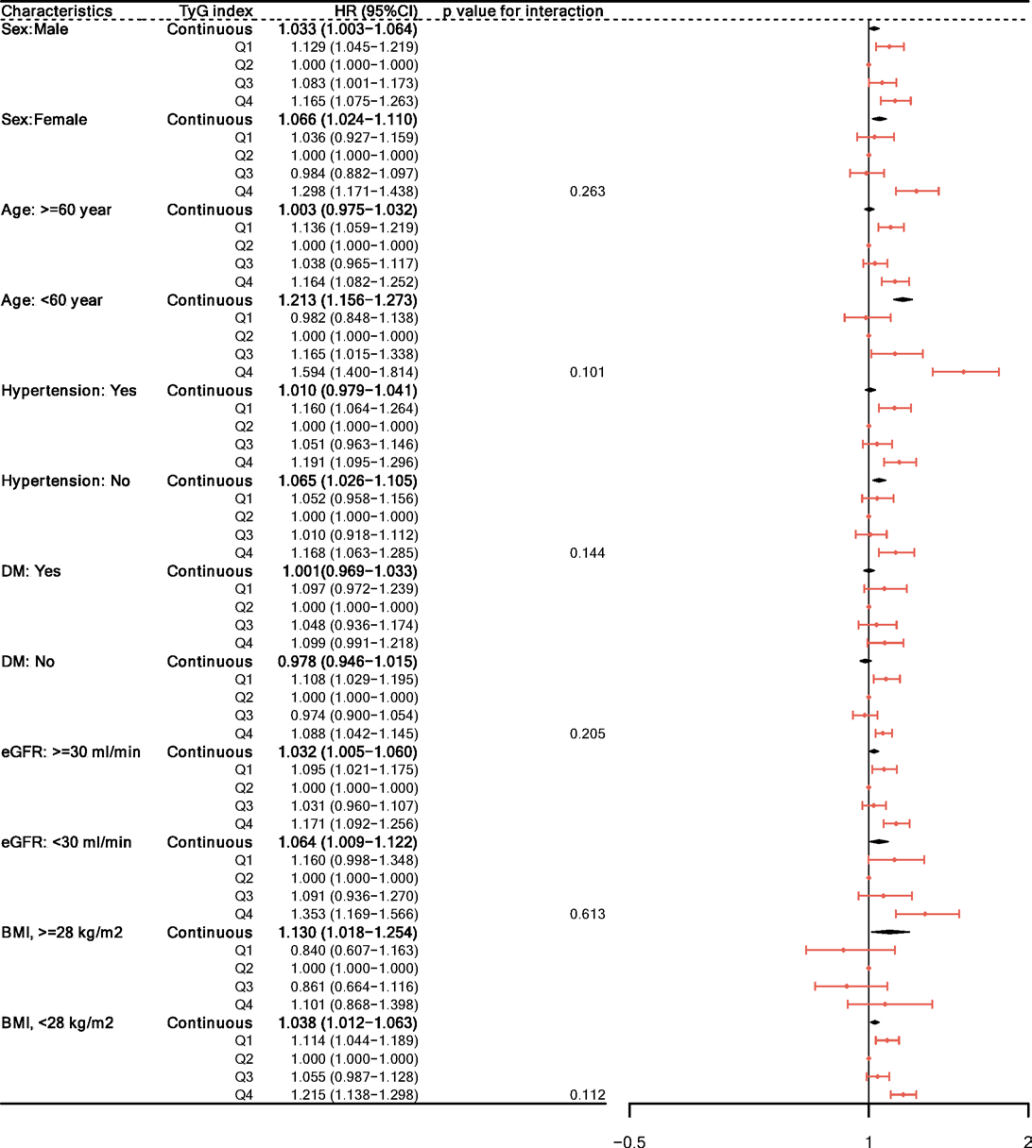
Subgroup analysis for MACE.

The association between the TyG index and risk of MACE was further evaluated across key clinical subgroups, as summarized in Table 3 and illustrated in Fig. 4.

**Table 3:** Subgroup analysis of the association between TyG index and MACE.

Characteristics	TyG index					*P*-interaction
	Continuous	Q1	Q2	Q3	Q4	
Sex						0.263
Male	1.033 (1.003–1.064) *	1.129 (1.045–1.219) **	Ref	1.083 (1.001–1.173) *	1.165 (1.075–1.263) ***	
Female	1.066 (1.024–1.110) **	1.036 (0.927–1.159)	Ref	0.984 (0.882–1.097)	1.298 (1.171–1.438) ***	
Age, years						0.101
≥ 60	1.003 (0.975–1.032)	1.136 (1.059–1.219) ***	Ref	1.038 (0.965–1.117)	1.164 (1.082–1.252) ***	
< 60	1.213 (1.156–1.273) ***	0.982 (0.848–1.138)	Ref	1.165 (1.015–1.338) *	1.594 (1.400-1.814) ***	
Hypertension						0.144
Yes	1.010 (0.979–1.041)	1.160 (1.064–1.264) ***	Ref	1.051 (0.963–1.146)	1.191 (1.095–1.296) ***	
No	1.065 (1.026–1.105) ***	1.052 (0.958–1.156)	Ref	1.010 (0.918–1.112)	1.168 (1.063–1.285) **	
DM						0.205
Yes	1.001(0.969–1.033)	1.097 (0.972–1.239)	Ref	1.048 (0.936–1.174)	1.099 (0.991–1.218)	
No	0.978 (0.946–1.015)	1.108 (1.029–1.195) **	Ref	0.974 (0.900-1.054)	1.128 (1.034–1.215) **	
eGFR, ml/min						0.613
≥ 30	1.032 (1.005–1.060) *	1.095 (1.021–1.175) *	Ref	1.031 (0.960–1.107)	1.171 (1.092–1.256) ***	
< 30	1.064 (1.009–1.122) *	1.160 (0.998–1.348)	Ref	1.091 (0.936–1.270)	1.353 (1.169–1.566) ***	
BMI, kg/m^2^						0.112
≥ 28	1.130 (1.018–1.254) *	0.840 (0.607–1.163)	Ref	0.861 (0.664–1.116)	1.101 (0.868–1.398)	
< 28	1.038 (1.012–1.063) **	1.114 (1.044–1.189) **	Ref	1.055 (0.987–1.128)	1.215 (1.138–1.298) ***	

DM: Diabetes mellitus, eGFR: Estimated glomerular filtration Rate, BMI: Body mass index.

Statistical Significance: *: *p* < 0.05, **: *p* < 0.01, ***: *p* < 0.001.

#### Sex

In male patients, both low (Q1, HR: 1.129, 95% CI: 1.045–1.219, *p* < 0.01) and high (Q4, HR: 1.165, 95% CI: 1.075–1.263, *p* < 0.001) TyG quartiles were associated with a significantly increased risk of MACE compared to Q2, whereas in females, only high TyG (Q4, HR: 1.298, 95% CI: 1.171–1.438, *p* < 0.001) was significant.

#### Age

Among patients aged ≥ 60 years, both Q1 (HR: 1.136, 95% CI: 1.059–1.219, *p* < 0.001) and Q4 (HR: 1.164, 95% CI: 1.082–1.252, *p* < 0.001) were significantly associated with increased MACE risk. In those < 60 years, only Q4 was significant (HR: 1.594, 95% CI: 1.400–1.814, *p* < 0.001).

#### Hypertension

In hypertensive patients, both Q1 (HR: 1.160, 95% CI: 1.064–1.264, *p* < 0.001) and Q4 (HR: 1.191, 95% CI: 1.095–1.296, *p* < 0.001) were associated with increased MACE risk. In non-hypertensive patients, only Q4 (HR: 1.168, 95% CI: 1.063–1.285, *p* < 0.01) was significant.

#### Diabetes mellitus

In patients without diabetes, both Q1 (HR: 1.108, 95% CI: 1.029–1.195, *p* < 0.01) and Q4 (HR: 1.128, 95% CI: 1.034–1.215, *p* < 0.01) were associated with MACE, while in those with diabetes, no significant associations were found across TyG quartiles.

Renal Function (eGFR):

For patients with eGFR ≥ 30 ml/min/1.73 m², Q1 (HR: 1.095, 95% CI: 1.021–1.175, *p* < 0.05) and Q4 (HR: 1.171, 95% CI: 1.092–1.256, *p* < 0.001) were significant; in those with eGFR < 30, only Q4 (HR: 1.353, 95% CI: 1.169–1.566, *p* < 0.001) was significant.

#### Body mass index (BMI)

Among participants with BMI < 28 kg/m^2^, both Q1 (HR: 1.114, 95% CI: 1.044–1.189, *p* < 0.01) and Q4 (HR: 1.215, 95% CI: 1.138–1.298, *p* < 0.001) were significantly associated with MACE. In those with BMI ≥ 28 kg/m^2^, only the continuous TyG index was significantly associated with MACE (HR: 1.130, 95% CI: 1.018–1.254, *p* < 0.05), and no significant differences were observed among quartiles.

There were no significant interactions between TyG index and subgroup variables (all p for interaction > 0.05). These findings suggest that the relationship between TyG index and MACE may be modified by sex, age, hypertension, diabetes status, renal function, and BMI.

### Sensitivity analysis

**Fig. 5:**
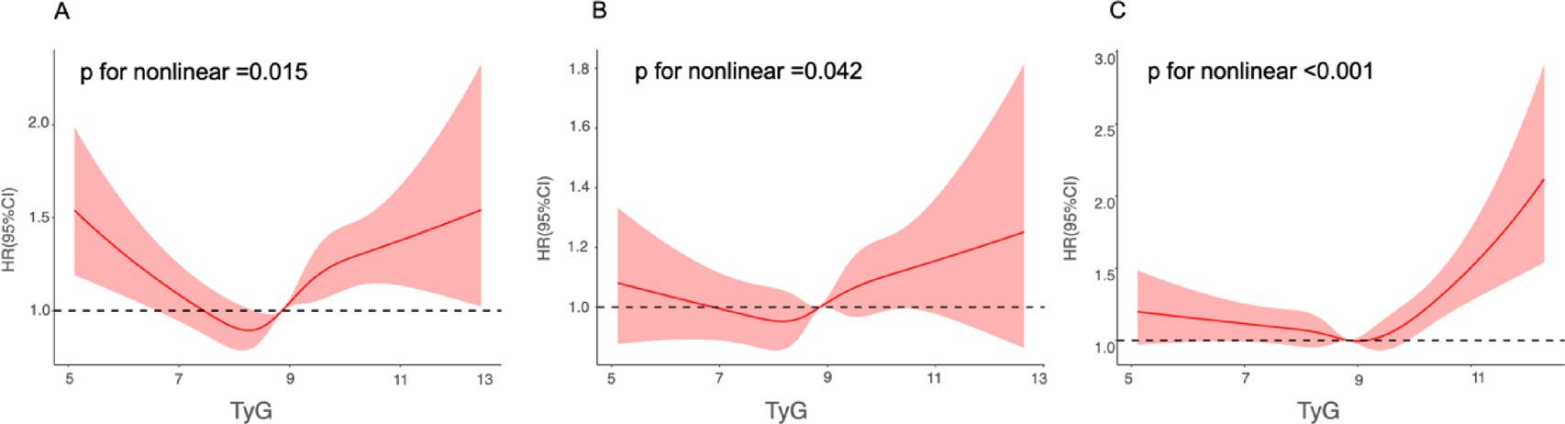
Restricted cubic spline curves illustrating the association between TyG and MACE across different follow-up periods. (**A**). Fully adjusted model in patients with follow-up duration within one year. (**B**). Fully adjusted model in patients with follow-up duration within three-year. (**C**). Fully adjusted model in patients with follow-up duration greater than three years. Hazard ratios are indicated by solid red lines and 95% CIs are indicated by shaded areas. CI, confidence interval; TyG, triglyceride-glucose index.

In the sensitivity analysis section of this study, we considered five aspects:

1. In model 3 (fully adjusted model), multivariate regression was used to adjust for the impact of multiple variables on the outcomes.
2. Patients were stratified by follow-up duration into three groups: within 1 year, within 3 years, and more than 3 years, and the association between TyG and MACE was calculated for each follow-up period using model 3 Cox regression, as shown in Table 4. In the overall follow-up period, the continuous TyG index was not significantly associated with MACE, whereas TyG quartiles Q1 and Q4 had HRs (95% CI) of 1.082 (1.018, 1.147), *p* < 0.05 and 1.123 (1.0537, 1.1920), *p* < 0.001, respectively, indicating significant associations with MACE. Quartile Q3 (HR: 1.044, 95% CI: 0.9787–1.1097) was not significantly associated with MACE (*p* > 0.05). In the 1-year and 2-year follow-up groups, the continuous TyG index and quartiles Q1 and Q3 (with Q2 as the reference) had p-values greater than 0.05, suggesting no significant association with MACE. Quartile Q4 had HRs (95% CI) of 1.167 (1.029–1.304) and 1.129 (1.026–1.233), respectively, with p-values < 0.05, indicating a significant association with MACE. In the “more than 3 years” follow-up group, the continuous TyG index and quartile Q3 (with Q2 as the reference) had p-values greater than 0.05, suggesting no significant association with MACE. Quartiles Q1 and Q4 had HRs (95% CI) of 1.099 (1.013–1.184) and 1.114 (1.021–1.207), respectively, with p-values < 0.05, indicating significant associations with MACE.
3. Restricted cubic spline (RCS) plots for TyG index and MACE were generated for different follow-up durations, as shown in Fig. 5. The p-values for nonlinearity in all follow-up time intervals were less than 0.05, suggesting a nonlinear association between the TyG index and MACE.
4. To assess the robustness of our results regarding missing data, we compared the findings from complete-case analyses with those obtained using multiple imputation for variables with less than 10% missingness. The associations between TyG quartiles (as well as TyG as a continuous variable) and the risk of MACE remained consistent across both analytic approaches. These findings suggest that the results are unlikely to be substantially influenced by the method used for handling missing data.

**Table 4:** Correlation between TyG Index and MACE in Populations Stratified by Follow-up Duration.

Duration	TyG index				
	Continuous	Q1	Q2	Q3	Q4
Overall	1.015 (0.988–1.042)	1.082 (1.018, 1.147) *	Ref	1.044 (0.9787, 1.1097)	1.123 (1.054, 1.192) ***
1-year	0.999 (0.949, 1.050)	1.043 (0.914, 1.172)	Ref	1.084 (0.955, 1.213)	1.167 (1.029, 1.304) *
2-year	1.028 (0.989, 1.068)	1.018 (0.920, 1.117)	Ref	1.085 (0.987, 1.183)	1.129 (1.026, 1.233) *
More than 2 years	1.020 (0.985, 1.055)	1.099 (1.013, 1.184) *	Ref	1.003 (0.915, 1.092)	1.114 (1.021, 1.207) *

Statistical Significance: *: *p* < 0.05, **: *p* < 0.01, ***: *p* < 0.001.

### Competing risk analysis

To address the potential impact of competing risks, we performed Fine-Gray subdistribution hazard analyses treating non-cardiovascular death as a competing event for MACE. The U-shaped association between TyG quartiles and MACE risk remained robust: compared with Q2, both Q1 (subdistribution HR: 1.15, 95% CI: 1.02–1.29, *p* = 0.021) and Q4 (subdistribution HR: 1.18, 95% CI: 1.08–1.29, *p* < 0.001) were significantly associated with higher risk of MACE, while Q3 was not significant. These findings indicate that the main results are not substantially affected by competing risk of non-cardiovascular death (see Supplementary Table 4).

## Discussion

This retrospective cohort study is the largest to date investigating the association between TyG levels and the risk of MACE in CKD patients. For the choice of endpoint events, we selected the commonly used four-point MACE composite, which includes acute myocardial infarction (AMI), hemorrhagic or ischemic stroke, cardiovascular-related death, and rehospitalization due to unstable angina or coronary revascularization surgery. In this study, we demonstrated a nonlinear relationship between TyG and MACE/all-cause mortality in CKD patients, suggesting that both excessively low and high TyG index levels may increase the risk of MACE and all-cause mortality. This finding was confirmed through survival analysis and sensitivity analyses of the outcomes.

For the selection of patients with impaired kidney function, we included individuals with CKD stages 3–4 (eGFR 15–60 ml/min/1.73 m^2^). As kidney function deteriorates, cardiovascular-related mortality and all-cause mortality significantly increase, especially in patients with an eGFR below 60 ml/min^21,22^. Patients with an eGFR less than 15 ml/min or those who have started dialysis are already in the terminal stage of the disease, with evident disturbances in the internal environment, electrolyte metabolism, and water overload, and many of these patients exhibit substantial vascular calcification. These patients tend to experience MACE much earlier than the general population^23^. Previous studies have investigated the 1-year MACE incidence in ESRD patients with different TyG index levels, indicating a significant association between TyG and MACE^24^. However, large-scale studies focusing on CKD stages 3–4 and the association between TyG and MACE are limited. Therefore, we chose CKD patients with eGFR values between 15 and 60 ml/min as the target population to identify clear risk factors and potential intervention targets for improving long-term outcomes.

CKD patients often exhibit significant lipid metabolism disorders, typically presenting as hypertriglyceridemia and low HDL-C levels^25^. In our study population, the average triglyceride level was 1.53 mmol/L, with 22.4% of patients showing high triglyceride levels. The average HDL-C level was 0.98 mmol/L, with 33.4% of patients having HDL-C levels below 0.9 mmol/L, consistent with the characteristic dyslipidemia of CKD^26,27^. The average TyG index level in our CKD population was 8.88, similar to levels reported in other studies, which range between 8.67 and 9.00^12,28^. Glucose and triglycerides, the two components of the TyG index, play crucial roles in the pathophysiological processes and complications of CKD. Glucose metabolism in CKD patients is affected by increased insulin resistance, impaired counter-regulatory hormone response (cortisol, growth hormone), malnutrition, and variability in exposure to oral hypoglycemic agents and exogenous insulin. ESRD patients often experience large fluctuations in blood glucose levels, with both hypoglycemia and hyperglycemia being common^29^. Both excessively high and low glucose levels may play important roles in the occurrence of all-cause mortality and cardiovascular events in CKD patients^30,31^. For triglyceride control in CKD, current research often suggests that elevated triglyceride levels are associated with poor renal or cardiovascular outcomes, with a general recommendation to maintain triglycerides below 1.7 mmol/L^32–34^. Large-scale cohort studies have also confirmed that lipid-lowering treatment can improve outcomes such as reducing the incidence of MACE^35^. However, triglyceride levels that are too low may be associated with malnutrition, inflammation, or other metabolic abnormalities, which could increase the risk of adverse outcomes in CKD patients. Therefore, the nonlinear relationship between the TyG index and MACE observed in our study is consistent with mainstream findings that both high and low levels of glucose and triglycerides are associated with adverse outcomes in CKD patients. The results of the stratified analyses by BMI and serum albumin further support the hypothesis that malnutrition may partly explain the elevated risk observed in the lowest TyG quartile (shown in Supplement Table 3). The U-shaped association was most evident among patients with lower BMI or hypoalbuminemia, both of which are established indicators of poor nutritional status and adverse outcomes in CKD.

In non-CKD individuals, the TyG index is a reliable surrogate for insulin resistance (IR). However, this association is confounded in CKD. Reduced lipoprotein lipase activity, impaired triglyceride clearance, and chronic inflammation result in a distinct dyslipidemia profile, which may weaken the association between TyG and insulin resistance compared to the general population^36^. This complex pathophysiology may help explain the non-linear, U-shaped relationship we observed, where both high (indicating IR) and low TyG levels (potentially reflecting malnutrition-inflammation) were associated with increased risk^36,37^.

The subgroup analyses in our study reveal important heterogeneity in the association between TyG index and the risk of major adverse cardiovascular events (MACE) among patients with CKD stages 3–4. Notably, both low and high TyG quartiles (Q1 and Q4) were associated with increased MACE risk in males, older adults (≥ 60 years), individuals with hypertension, and those without diabetes, suggesting a possible U-shaped relationship in these populations. In contrast, a high TyG index (Q4) was the predominant risk factor for MACE in females, younger patients (< 60 years), non-hypertensive, diabetic, and obese individuals (BMI ≥ 28 kg/m²).

These findings highlight that the prognostic value of the TyG index may be modified by demographic and clinical characteristics and suggest that both metabolic derangements and malnutrition could contribute to adverse cardiovascular outcomes in certain subgroups. For example, the presence of a U-shaped association in non-diabetic or non-obese populations may reflect the dual impact of insulin resistance and protein-energy wasting, both of which are prevalent in CKD. This is consistent with previous studies reporting that TyG index is a stronger predictor of cardiovascular risk in non-diabetic cohorts^38^and in those with preserved nutritional status.

Our secondary outcome analysis showed a U-shaped association between TyG index and all-cause mortality, similar to the findings for MACE. Both low and high TyG quartiles were associated with increased mortality compared to the reference group. Interestingly, the continuous TyG index was inversely associated with mortality in the fully adjusted model, suggesting that low TyG—potentially reflecting malnutrition or protein-energy wasting—may play a prominent role in mortality risk among patients with advanced CKD^39,40^. These results underscore the need to consider both metabolic and nutritional factors when evaluating prognosis in this population.

Given that the primary outcome of the study was MACE, we also included the AIP index. In univariate and multivariate analyses (in which AIP was included without HDL-c and TG), the HR for AIP was less than 1 in Cox regression for MACE, which contradicts prior findings that generally consider AIP a risk factor for MACE^41,42^. The predictive role of AIP for cardiovascular events in patients with moderate-to-severe CKD remains to be further investigated.

### Limitations

Several limitations should be acknowledged. First, as a retrospective study, causality cannot be established, and the overall level of evidence is inherently limited. Second, the lack of systematic albuminuria data for all participants precluded the use of both eGFR and albuminuria for comprehensive CKD classification and risk stratification. Third, comprehensive markers of nutritional status—such as subjective global assessment (SGA) or malnutrition-inflammation score—were not available, and this absence of more detailed nutritional assessment may result in residual confounding. Future studies should incorporate more comprehensive nutritional evaluation tools. Fourth, although multivariate Cox regression was used to adjust for a wide range of confounders, our approach to confounder selection was partially based on statistical criteria (univariate *p* < 0.10), which may not fully account for all clinically relevant confounders or causal relationships. Propensity score matching (PSM) or other advanced methods might help to further mitigate selection bias. Fifth, patients with a prior history of MACE were excluded to focus on first events; however, this may have resulted in selection bias by removing higher-risk individuals from the cohort. Sixth, we did not establish a specific optimal TyG range or evaluate interventions to modify TyG, so the clinical implications of our findings should be interpreted with caution and require further validation. Finally, ascertainment of MACE events may be incomplete, as some CKD patients might have had unrecorded events due to delayed or absent medical attention or because they sought care outside the CRDS network, potentially introducing information bias.

## Conclusion

This large-scale retrospective cohort study demonstrated a nonlinear relationship between the TyG index and the risk of MACE and all-cause mortality in CKD stages 3–4 patients. Subgroup analyses indicated that age, diabetes status, hypertension, and BMI influenced the relationship between TyG and MACE, highlighting the need for tailored risk management strategies in CKD populations.

### Statistical analysis

Patients were divided into quartiles based on TyG index values. Depending on the distribution type, continuous variables were displayed as mean ± standard deviation or median and interquartile range (25th and 75th percentile). Differences between groups were assessed using ANOVA or the Kruskal–Wallis H test, depending on whether the distribution was normal. Categorical variables were expressed as numbers and proportions, and comparisons were made using either the Chi-square test or Fisher’s exact test, as appropriate. The Kaplan–Meier (KM) curves were used to describe survival across different TyG quartiles, and the log-rank test was used to determine group differences. Univariate and multivariate Cox regression models were employed to explore the relationship between the TyG index and MACE. Model 1 was unadjusted for TyG, whereas Model 2 included age and gender as covariates; ethnic adjustment was not performed as the cohort consisted of a homogeneous population. In all multivariate Cox regression analyses (Model 3), we included adjustment for medication use (insulin, lipid-lowering agents, RAS inhibitors, and diuretics), along with age, sex, blood pressure, BMI, comorbidities, and relevant laboratory variables. and included variables with a p-value of less than 0.10 in the univariate Cox regression analysis (for MACE occurrence). Missing values were handled using multiple imputation by chained equations (MICE). Variables with more than 10% missing data were excluded from analysis, while those with less than 10% missing data were imputed using MICE. Restricted cubic spline (RCS) plots were used within the Cox regression models to assess the nonlinear relationship between the TyG index and primary outcomes. The number and placement of knots for the RCS were determined based on standard methodological recommendations and commonly used R packages. In the primary analysis, five knots were placed at the 5th, 25th, 50th, 75th, and 95th percentiles of the TyG index distribution. Sensitivity analyses using models with four and six knots were also performed to confirm the robustness of the results (see Supplementary Tables 2 and Supplementary Fig. 3).

Two-sided p-values less than 0.05 were considered statistically significant. Statistical analyses were performed using R software, version 4.2.1. Data analysis employed the R packages mice, dplyr, survminer, ggplot2, survival, tidyr, foreign, and rms.

## Supplementary Information

Below is the link to the electronic supplementary material.

Supplementary Material 1

Supplementary Material 2

## Data Availability

Data Availability StatementThe data used in this study were obtained from the China Renal Data System (CRDS), a restricted-access database. Due to data privacy regulations and institutional policies, the dataset cannot be shared publicly. However, researchers may apply for access to the CRDS database through the appropriate institutional review board and data use agreement. For further information regarding data access, please contact the first author, Fan Zhu (colinzhu1991@gmail.com).
