# Supplementary material for: Association between the triglyceride-glucose index and major adverse cardiovascular events in patients with chronic kidney disease stages 3–4
Source: Sci Rep. 2025 Aug 5;15:28538. doi: 10.1038/s41598-025-14057-1 (PMC12325913; doi:10.1038/s41598-025-14057-1)
Supplement: Supplementary file 1 — Supplementary Material 1 [file 41598_2025_14057_MOESM1_ESM.docx]

**Supplementary Table 1.** Sensitivity Analysis of the Association Between TyG Index and MACE in CKD Stages 3–4: Complete-Case Analysis vs. Multiple Imputation

| **Exposure** | **Reference** | **Model** | **n** | **Events** | **HR (95% CI) Q1** | **HR (95% CI) Q3** | **HR (95% CI) Q4** | **HR (95% CI) per 1-unit TyG** | **P for trend** |
| --- | --- | --- | --- | --- | --- | --- | --- | --- | --- |
| TyG Quartiles | Q2 | Complete case | 40,120 | 6,650 | 1.08 (1.01–1.16) | 1.05 (0.98–1.12) | 1.13 (1.06–1.22) | — | 0.001 |
| TyG Quartiles | Q2 | Multiple imputation | 48,935 | 7,775 | 1.04 (1.01–1.15) | 1.12 (0.92–1.21) | 1.17 (1.09–1.20) | — | 0.001 |
| TyG (continuous) | — | Complete case | 40,120 | 6,650 | — | — | — | 1.10 (1.05–1.15) | <0.001 |
| TyG (continuous) | — | Multiple imputation | 48,935 | 7,775 | — | — | — | 1.13 (1.07–1.18) | <0.001 |

**Supplementary Table 2.** Sensitivity Analysis of the Nonlinear Association Between TyG Index and MACE Using Different Numbers of Knots in Restricted Cubic Spline (RCS) Models

| **Number of Knots** | **Knots Placement (percentiles)** | **P for Nonlinearity** | **Overall Curve Shape** | **HR (Q4 vs Q2, 95% CI)** | **HR (Q1 vs Q2, 95% CI)** |
| --- | --- | --- | --- | --- | --- |
| 4 | 5th, 35th, 65th, 95th | 0.035 | U-shaped | 1.04 (1.01–1.11) | 1.07 (1.03–1.19) |
| 5 | 5th, 25th, 50th, 75th, 95th | 0.009 | U-shaped | 1.12 (1.05–1.20) | 1.08 (1.01–1.15) |
| 6 | 5th, 23rd, 41st, 59th, 77th, 95th | 0.018 | U-shaped | 1.09 (1.03–1.18) | 1.05 (1.02–1.15) |

**Supplementary Table 3.** Association Between TyG Quartiles and MACE Risk Stratified by Nutritional Status (BMI and Albumin)

| **Subgroup** | **TyG Quartile** | **HR (95% CI)** | **P-value** |
| --- | --- | --- | --- |
| **BMI < 18** | Q1 | 1.19 (1.05–1.34) | 0.004 |
|  | Q2 (ref) | 1.00 (reference) | — |
|  | Q3 | 1.05 (0.93–1.17) | 0.41 |
|  | Q4 | 1.21 (1.09–1.35) | 0.001 |
| **BMI ≥ 18** | Q1 | 1.07 (0.97–1.18) | 0.17 |
|  | Q2 (ref) | 1.00 (reference) | — |
|  | Q3 | 1.03 (0.93–1.14) | 0.54 |
|  | Q4 | 1.10 (1.01–1.21) | 0.035 |
| **Albumin < 30** | Q1 | 1.23 (1.08–1.41) | 0.002 |
|  | Q2 (ref) | 1.00 (reference) | — |
|  | Q3 | 1.07 (0.94–1.22) | 0.31 |
|  | Q4 | 1.28 (1.13–1.45) | <0.001 |
| **Albumin ≥ 30** | Q1 | 1.06 (0.95–1.19) | 0.28 |
|  | Q2 (ref) | 1.00 (reference) | — |
|  | Q3 | 1.02 (0.91–1.14) | 0.74 |
|  | Q4 | 1.09 (0.98–1.21) | 0.11 |

**Supplementary Table 4.** Association Between TyG Quartiles and MACE Risk Using Fine-Gray Competing Risk Model (Non-cardiovascular Death as Competing Event)

| **TyG Quartile** | **Subdistribution HR (95% CI)** | **P-value** |
| --- | --- | --- |
| Q1 | 1.15 (1.02–1.29) | 0.021 |
| Q2 (ref) | 1.00 (reference) | — |
| Q3 | 1.04 (0.96–1.13) | 0.32 |
| Q4 | 1.18 (1.08–1.29) | <0.001 |
