# Supplementary material for: Association between the triglyceride-glucose index and major adverse cardiovascular events in patients with chronic kidney disease stages 3–4
Source: Sci Rep. 2025 Aug 5;15:28538. doi: 10.1038/s41598-025-14057-1 (PMC12325913; doi:10.1038/s41598-025-14057-1)
Supplement: Supplementary file 2 — Supplementary Material 2 [file 41598_2025_14057_MOESM2_ESM.docx]

**Supplementary Figures**

**
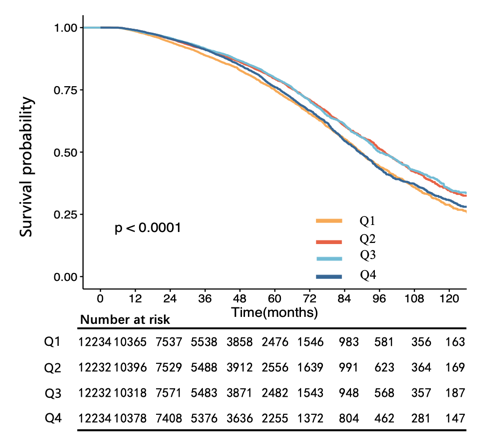
**

**Supplementary Figure 1.** Kaplan–Meier curves illustrating survival probability for major adverse cardiovascular events (MACE) according to TyG index quartiles (Q1–Q4).


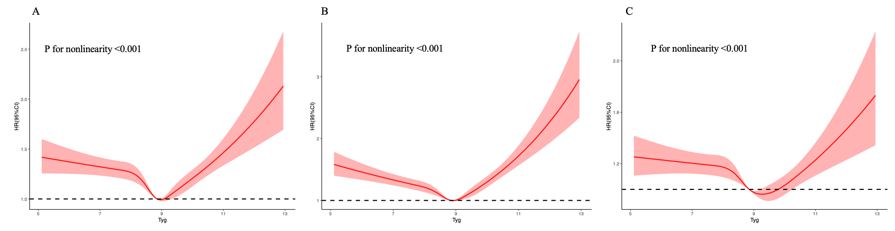


**Supplementary Figure 2.** Restricted cubic spline (RCS) curves depicting the association between the TyG index and all-cause mortality in three Cox regression models: Model 1 (unadjusted, **Panel A**), Model 2 (adjusted for age and sex, **Panel B**), and Model 3 (fully adjusted, **Panel C**).

**
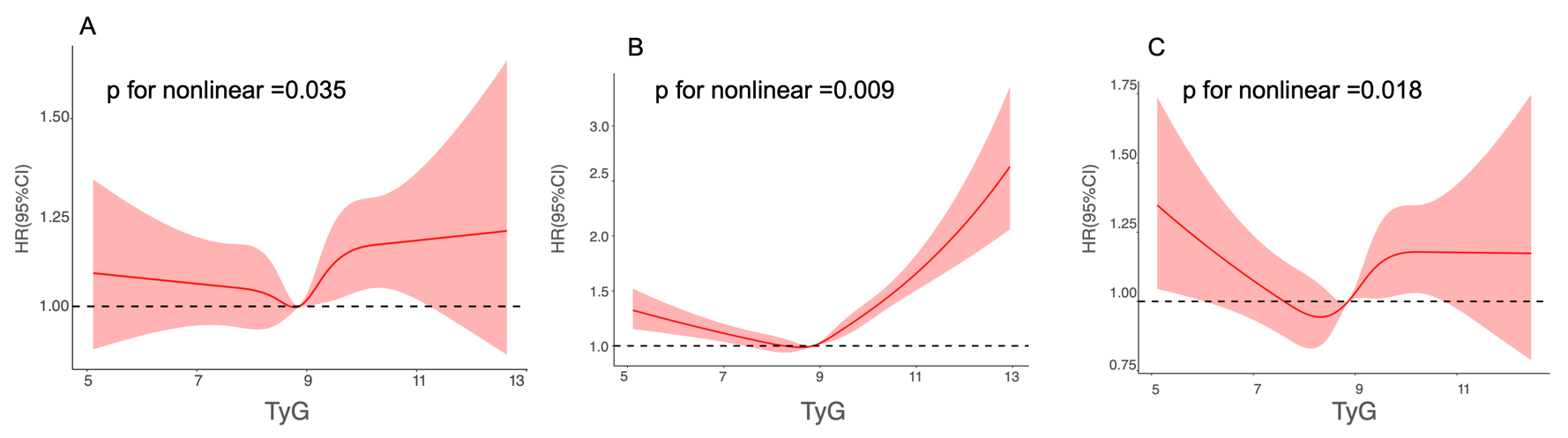
**

**Supplementary Figure 3.** Restricted cubic spline (RCS) curves showing the nonlinear association between TyG index and risk of major adverse cardiovascular events (MACE). **Panel A**, 4 knots; **Panel B**, 5 knots; **Panel C**, 6 knots.
